# Retinal diseases in a reference center from a Western Amazon capital city

**DOI:** 10.1590/S1679-45082015AO3538

**Published:** 2015

**Authors:** Fernando Korn Malerbi, Nilson Hideo Matsudo, Adriano Biondi Monteiro Carneiro, Claudio Luiz Lottenberg

**Affiliations:** 1 Hospital Israelita Albert Einstein, São Paulo, SP, Brazil.; 2 Instituto Israelita de Responsabilidade Social, Hospital Israelita Albert Einstein, São Paulo, SP, Brazil.

**Keywords:** Diabetic retinopathy, Telemedicine, Retinal detachment, Cataract, Blindness

## Abstract

**Objective:**

To describe retinal diseases found in patients who were waiting for treatment at a tertiary care hospital in Rio Branco, Acre, Brazil*. *

**Methods:**

Patients underwent slit lamp biomicroscopy, dilated fundus exam and ocular ultrasound. Patients were classified according to phakic status and retinal disease of the most severely affected eye.

**Results:**

A total of 138 patients were examined. The mean age was 51.3 years. Diabetes was present in 35.3% and hypertension in 45.4% of these patients. Cataract was found in 23.2% of patients, in at least one eye. Retinal examination was possible in 129 patients. The main retinal diseases identified were rhegmatogenous retinal detachment (n=23; 17.8%) and diabetic retinopathy (n=32; 24.8%). Out of 40 patients evaluated due to diabetes, 13 (32.5%) had absent or mild forms of diabetic retinopathy and did not need further treatment, only observation.

**Conclusion:**

Diabetic retinopathy was the main retinal disease in this population. It is an avoidable cause of blindness and can be remotely evaluated, in its initial stages, by telemedicine strategies. In remote Brazilian areas, telemedicine may be an important tool for retinal diseases diagnosis and follow-up.

## INTRODUCTION

Brazil is a continent-sized country with very heterogeneous socioeconomic realities. In 2013, Brazil had one ophthalmologist to 11,604 inhabitants, *i.e.*, more than what has been recommended by the World Health Organization (WHO). However, the distribution of these specialists is uneven, and the North Region is the most challenged: of the 17,3 one thousand ophthalmologists in Brazil, only 557 (3%) work in this area, and the ratio is one ophthalmologist to 30,491 inhabitants. The census of the *Conselho Brasileiro de Oftalmologia* (CBO) [Brazilian Council of Ophthalmology]^(1)^ identified only 20 ophthalmologists in the State of Acre, in 2013, a ratio of one ophthalmologist to 38,823 inhabitants. Rio Branco (AC) was considered the third capital city with the lowest density of ophthalmologists per inhabitant in the country, after Macapá (AP) and Boa Vista (RR).^(1,2)^

Retinal diseases are the most common cause of blindness in adults in Brazil urban populations.^(3)^ The main retinal diseases include diabetic retinopathy (DR), which has been associated with low vision or blindness in 38.7% of affected individuals, in a national study.^(4)^ DR, although preventable, is the leading cause of blindness in the working population, and its prevalence in Brazil was estimated at 7.6 to 39% of individuals with diabetes. This marked variation in prevalence can be explained by differences in the methods used to diagnose diabetes in the studies available, and due to underdiagnosed cases of the condition.^(4)^

A Brazilian population-based study carried out to investigate the causes of blindness in urban adults showed that retinal diseases are the main cause of blindness, followed by cataracts and glaucoma. In addition to DR, in our country, age-related macular degeneration and retinal detachment are important causes of retinal blindness.^(3)^ Macular degeneration occurs primarily in patients aged over 60 years, and may present as “dry” or exudative disease; it usually leads to central scotoma. Retinal detachment, if not treated early, can cause permanent loss of the central and peripheral visions.

Locations where specialists are scarce usually have a yet smaller number of subspecialists. Thus, in the first half of 2014, in Rio Branco (AC), there was a large number of patients with retinal diseases awaiting out-of-home treatment (OHT). OHT is a legal instrument established by the Ministry of Health that guarantees, under the Unified Public Health System (SUS – *Sistema Único de Saúde*), medical treatment for patients with diseases that cannot be treated in their city of residence and/or requiring medical care with elective high- and medium-complexity procedures, after all treatment options in their place of residence have been exhausted. The program provides medical appointments; previously scheduled outpatient, inpatient and/or surgical treatment; return transportation tickets for the patients to go to the place where treatment will be conducted and back to their place of residence; and allowances for meals and lodging.^(5)^

Telemedicine has been successfully applied for monitoring of DR,^(6-8)^ meeting the growing demand of diabetic patients. Digital retinal images can be performed by trained non-medical personnel, and submitted to a center for review by an expert physician.

## OBJECTIVE

To describe the retinal diseases found in patients awaiting out-of-home treatment at a tertiary care hospital in Rio Branco, Acre, Brazil.

## METHODS

This study was based on data from patients awaiting OHT for retinal diseases. These patients had been previously examined by ophthalmologists from the *Fundação Hospitalar Estadual do Acre* (FUNDHACRE), a tertiary care hospital in Rio Branco (AC), where patients from all over the State were referred to. An ophthalmologist specialized in retina and an ophthalmic technologist from *Hospital Israelita Albert Einstein* traveled to Rio Branco (AC), in April 2014, for this evaluation.

The project was submitted to the Research Ethics Committee of the *Hospital Israelita Albert Einstein*, as per Resolution 466/2012, and since it was considered related to management studies and process improvement, it did not require approval by the committee.

The data analysis took into account some demographic information - sex and age, as well as clinical information, such as presence of *diabetes mellitus* and hypertension. Ophthalmic data were analyzed according to the following examinations: slit lamp biomicroscopy, mydriatric fundus exam by binocular indirect ophthalmoscopy, fundus biomicroscopy (in selected cases) and ocular ultrasound (in cases of media opacity).

The biomicroscopy evaluated the state of the crystalline lens or the presence of intraocular lenses, as well as cases of crystalline lens subluxation.

The dilated fundus exam evaluated the optic disc and retina, in both posterior pole and periphery. DR, when present, was classified as nonproliferative or proliferative and evaluated for the presence of diabetic macular edema.

The retinal evaluation described was based on the eye with the most severe retinal changes. In patients with more than one retinal abnormality, the most severe was described, or the most threatening to the vision.

According to the examination protocol, when retinal examination was precluded by media opacity, we tested for the presence of light perception in the eye examined. If the examined eye had no light perception, the investigation was stopped for that eye.

## RESULTS

We evaluated data from 138 patients. These patients were aged 51.3±18.0 years (mean, standard deviation), and 75 patients were male (54.4%). Diabetes was reported by 49 patients (35.5%) and hypertension by 67 patients (45.5%).

### Cataract

Forty-two patients had transparent crystalline lenses in both eyes (30.4%), and 50 had at least one pseudophakic eye (36.2%). Cataract was present in at least one eye, in 32 patients (23.2%). Congenital cataract was diagnosed in two patients (both eyes), and one patient had subluxated crystalline lenses in both eyes. Two patients had aphakia (one patient in both eyes), and one patient had an ocular prosthesis in one eye. Seven patients could not undergo anterior chamber examination in at least one eye due to media opacity, phthisis bulbi or neovascular glaucoma with disorganized anterior chamber (one patient could not be examined in both eyes).

### Retinal evaluation

Six patients were unable to undergo retinal examination due to media opacity and had no light perception in the examined eye. These patients were excluded from further analysis. Three other patients had retinal examination precluded by media opacity, but there was light perception, and these patients underwent ultrasonography, which revealed attached retinas and no detectable abnormalities. These patients were also excluded from further analysis.

For the 129 remaining patients, fundoscopic findings were distributed as shown on table 1.

**Table 1:** Fundoscopic changes in patients awaiting out-of-home treatment in Rio Branco, Acre, April 2014

Change	Number of patients	Observation
Chorioretinal scar	6	-
Retinal dystrophy	3	-
Age-related macular degeneration	5	1 exudative case with indication for treatment
Rhegmatogenous retinal detachment	23	-
Postoperative management of retinal detachment	10	6 eyes with silicone oil
Glaucoma	14	1 case of congenital glaucoma
Vitreous hemorrhage	1	Not associated with diabetes
Undetermined maculopathy	4	-
Epiretinal membrane	3	-
Ischemic optic neuropathy	1	Sequela
Choroidal nevus or tumor	2	-
Normal exam	20	Of these, 8 patients with diabetes
Diabetic retinopathy	32	10 non-proliferative, of which 5 with macular edema; the other 22 cases were proliferative
Hypertensive retinopathy	3	2 with retinal vascular occlusion
Posterior uveitis	2	-

Total	129	

## DISCUSSION

Out of 138 patients awaiting OHT for retinal diseases who were examined, 40 had evaluations ordered due to DR (28.9%). Of these, 32 had retinal changes and 8 had normal retinal exams.

As for the crystalline lens evaluation, 36.2% of patients had pseudophakia in at least one eye. This piece of data indicates that a significant number of patients had undergone high-complexity eye treatments, such as cataract extraction with intraocular lens implantation, despite the small number of ophthalmologists in the region.

Among the retinal diseases, the two leading causes of retinal changes were rhegmatogenous retinal detachment and DR. These findings are in agreement with literature data that also report the two diseases as major causes of retinal blindness in adults from urban populations.^(3)^

Rhegmatogenous retinal detachment was present in 23 patients (17.8%). Ten patients were in the late postoperative period after surgery for retinal detachment repair, and required follow up with a retina subspecialist; 6 of these patients had silicone oil in the vitreous cavity and were candidates for further surgery to remove it.

Diabetic retinopathy was present in 32 patients (24.8%), 27 of which required treatment for proliferative DR or macular edema.

Other retinal disorders such as chorioretinitis scars, dystrophies, age-related macular degeneration, undiagnosed macular diseases (“further clarification needed”), epiretinal membrane, vitreous hemorrhage, posterior uveitis, retinal vascular occlusion, hypertensive retinopathy, and choroidal diseases were present in 29 patients.

DR is a preventable disease that can be monitored remotely in early stages. Screening since early stages of *diabetes mellitus* reduces the need for the treatment of DR complications.^(9)^ Twenty patients who were awaiting OHT did not present any retinal changes; 8 out of these 20 had diabetes. Diabetic maculopathy can be suspected in patients with diabetes and poor visual acuity, and patients with these characteristics must often undergo further tests, such as fluorescein angiography and optical coherence tomography to detect subclinical abnormalities.^(10,11)^

Thirteen (32.5%) out of the 40 patients assessed for diabetes required monitoring only (cases of absent DR, or nonproliferative DR with no macular edema) and 27 (67.5%) patients required treatment due to macular edema or proliferative DR.

Brazil has an estimated 11.9 million people with diabetes.^(12)^ Data from the literature suggest that, after 15 years of disease, approximately 78% of patients with diabetes have some degree of DR.^(13)^

From the health economics perspective, the prevention of DR has been established as more cost-effective than the treatment of this complication. Some studies show that the amount spent for a patient with advanced DR is more than ten times greater than the cost to treat it at early stages; the cost increases with the progression of the disease.^(14)^ One example is the data calculated for the German health system, in 2002, according to which the amount that could have been saved with prevention of advanced DR accounts to 1.5% of total health care expenses.^(14)^

In Brazil, due to the large size of the country and the poor distribution of specialists, the cost is supposed to be even greater, considering the cost of OHT.

Monitoring of patients with diabetes and absent or early-stage retinopathy can be carried out via telemedicine, with a good cost-effectiveness profile.^(13,15-18)^ Such a strategy can help meet the demand for subspecialists in remote locations, providing support in treatment-related decision-making and treatment follow-up.

Telemedicine is included in the recommendations for Brazilian ophthalmology proposed by the CBO.^(2)^ In the patient sample for this study, all patients awaiting OHT could have benefited from remote access to an expert opinion. In this way, patients with normal retinal examinations could reschedule their follow-up locally, avoiding travel, and patients requiring more complex treatment approaches could arrange their travel and treatment with more urgency.

We believe that, in order to reduce cases of avoidable blindness resulting from retinal diseases, in remote areas of Brazil, some measures can be taken, such as: (i) career planning to attract and retain specialists in remote areas; (ii) investment in pieces of equipment for diagnosis and treatment of retinal diseases (retinal cameras, retinal tomography, photocoagulation devices, operating room, etc.); (iii) capacity building and training of ophthalmologists to diagnose and treat retinal diseases; (iv) implementing a telemedicine system capable of sending images for remote diagnosis and counseling by subspecialists in reading centers; (v) capacity building and training of professionals for health education, as well as performance and delivery of retinal images for the telemedicine strategy.

## CONCLUSION

The main retinal diseases in this group of patients were diabetic retinopathy and retinal detachment. Diabetic retinopathy is a preventable cause of blindness, which can be remotely monitored at early stages, through telemedicine strategies. In remote Brazilian locations, telemedicine can be an important tool for monitoring of retinal diseases.
