# Supplementary material for: Retinal diseases in a reference center from a Western Amazon capital city
Source: Einstein (Sao Paulo). 2015 Oct-Dec;13(4):530–4. doi: 10.1590/S1679-45082015AO3538 (PMC4878626; doi:10.1590/S1679-45082015AO3538)
Supplement: Translation

# Translation: Doenças retinianas em um centro de referência de capital estadual na Amazônia Ocidental

**Authors:** Fernando Korn Malerbi, Nilson Hideo Matsudo, Adriano Biondi Monteiro Carneiro, Claudio Luiz Lottenberg

**Affiliations:** 1 Hospital Israelita Albert Einstein, São Paulo, SP, Brasil.; 2 Instituto Israelita de Responsabilidade Social, Hospital Israelita Albert Einstein, São Paulo, SP, Brasil.

**Keywords:** Retinopatia diabética, Telemedicina, Descolamento retiniano, Catarata, Cegueira

## Abstract

**Objetivo:**

Descrever as doenças de retina encontradas em pacientes que aguardavam tratamento para doenças retinianas em um hospital terciário de Rio Branco, Acre, Brasil.

**Métodos:**

Foi realizado um exame oftalmológico compreendendo biomicroscopia com lâmpada de fenda, mapeamento de retina e ultrassonografia ocular. Os pacientes foram classificados de acordo com o *status* fácico e a doença retiniana que acometia o olho de maior gravidade.

**Resultados:**

Foram atendidos 138 pacientes. A média de idade foi de 51,3 anos. A distribuição de diabetes foi de 35,3% e de hipertensão arterial foi de 45,5%. Catarata esteve presente em pelo menos um dos olhos em 23,2%. Tiveram o exame da retina possibilitado 129 pacientes. As principais doenças retinianas detectadas foram descolamento de retina regmatogênico (n=23; 17,8%) e retinopatia diabética (n=32; 24,8%). Dos 40 pacientes avaliados em função do diabetes, 13 (32,5%) apresentavam retinopatia ausente ou estágios iniciais de retinopatia e necessitavam apenas de acompanhamento.

**Conclusão:**

A principal doença retiniana foi a retinopatia diabética, uma causa de cegueira evitável que pode ser acompanhada à distância, nos estágios iniciais, por meio de estratégias de telemedicina. A telemedicina pode ser uma importante ferramenta no acompanhamento de doenças retinianas em localidades remotas no Brasil.

## INTRODUÇÃO

O Brasil é um país de dimensões continentais e que abriga realidades socioeconômicas muito heterogêneas. Em 2013, o Brasil contava com um oftalmologista para cada 11.604 habitantes, ou seja, em número acima do preconizado pela Organização Mundial da Saúde (OMS). No entanto, a distribuição destes especialistas é desequilibrada, sendo a Região Norte a mais desfavorecida: dos 17,3 mil oftalmologistas que atuam no Brasil, apenas 557 (3%) atuavam na Região Norte, o que corresponde a um oftalmologista para cada 30.491 habitantes. O censo do Conselho Brasileiro de Oftalmologia (CBO)^(1)^ identificou apenas 20 oftalmologistas atuando no Estado do Acre, em 2013, o que correspondia a uma proporção de um oftalmologista para cada 38.823 habitantes. Rio Branco (AC) foi considerada a terceira capital de Estado com a menor densidade de oftalmologistas por habitante do país, encontrando-se atrás apenas de Macapá (AP) e Boa Vista (RR).^(1,2)^

Doenças da retina são a causa mais comum de cegueira em adultos em populações urbanas no Brasil.^(3)^ Entre as principais doenças da retina, estão a retinopatia diabética (RD), que foi associada à baixa visão ou à cegueira em 38,7% dos indivíduos acometidos, em um estudo nacional.^(4)^ A RD, uma causa evitável de cegueira, é principal causa de cegueira na população economicamente ativa, e sua prevalência no Brasil foi estimada entre 7,6 a 39% dos indivíduos com diabetes. Essa grande variação na prevalência é explicada por diferenças na metodologia empregada para o diagnóstico de diabetes nos diversos estudos disponíveis e por causa do subdiagnóstico de diabetes.^(4)^

Estudo populacional brasileiro realizado para investigar causas de cegueira em adultos da área urbana revelou que as doenças de retina correspondem à principal causa de cegueira, seguidas por catarata e glaucoma. Além da RD, em nosso meio, a degeneração macular relacionada à idade e o descolamento de retina são importantes causas retinianas de cegueira.^(3)^ A degeneração macular ocorre principalmente em pacientes com idade superior a 60 anos, nas formas “seca” ou exsudativa, geralmente causando escotoma central. O descolamento de retina, se não tratado precocemente, pode causar uma perda definitiva da visão central e periférica.

Áreas com escassez de especialistas apresentam uma ainda escassez maior de subespecialistas. Assim, no primeiro semestre de 2014, havia em Rio Branco (AC), uma grande demanda de pacientes com doenças de retina que aguardavam tratamento fora do domicílio (TFD). O TFD é um instrumento legal instituído pelo Ministério da Saúde que garante, no âmbito do Sistema Único de Saúde (SUS), o tratamento médico para pacientes portadores de doenças não tratáveis no município de residência e/ou que necessitem de assistência médico-hospitalar com procedimentos considerados de alta e média complexidade eletiva, desde que esgotadas todas as formas de tratamento de saúde na localidade em que o paciente residir. Compete a esse programa oferecer: consulta; tratamento ambulatorial, hospitalar e/ou cirúrgico previamente agendados; passagens de ida e volta para que o paciente possa deslocar-se até o local onde será realizado o tratamento e retornar à sua cidade de origem; e ajuda de custo para alimentação e hospedagem.^(5)^

A telemedicina tem sido empregada com sucesso no rastreamento da RD,^(6-8)^ possibilitando atender à demanda crescente de pacientes com diabetes. O exame de retinografia digital pode ser realizado por profissional não médico treinado e enviado a centro de leitura, no qual é avaliado por médico especialista.

## OBJETIVO

Descrever as doenças de retina encontradas em pacientes que aguardavam tratamento fora do domicílio em um hospital terciário de Rio Branco, Acre, Brasil.

## MÉTODOS

Este estudo baseou-se na análise de dados de pacientes que aguardavam TFD por doenças retinianas. Esses pacientes já tinham sido previamente examinados por oftalmologistas da Fundação Hospitalar Estadual do Acre (FUNDHACRE), um hospital terciário de Rio Branco (AC), que recebia encaminhamentos de todo o Estado. Um oftalmologista especialista em retina e um tecnólogo oftálmico da equipe do Hospital Israelita Albert Einstein deslocaram-se até Rio Branco (AC), em abril de 2014 para a avaliação.

O projeto foi submetido ao Comitê de Ética em Pesquisa do Hospital Israelita Albert Einstein, conforme resolução 466/2012, que considerou tal projeto relacionado a estudos de gestão e melhorias de processos, sendo isento de avaliação pelo comitê.

A análise dos dados levou em conta informações demográficas, tais como sexo e idade, e informações clínicas, como presença de *diabetes mellitus* e hipertensão arterial. Os dados oftalmológicos foram analisados de acordo com os seguintes exames realizados: biomicroscopia em lâmpada de fenda, mapeamento de retina sob midríase por oftalmoscopia binocular indireta, biomicroscopia de fundo (em casos selecionados) e ultrassonografia ocular (em casos de opacidade de meios).

O exame de biomicroscopia avaliou o estado do cristalino ou a presença de lente intraocular, e casos de subluxação cristaliniana.

O exame de mapeamento de retina avaliou o disco óptico e a retina, tanto no polo posterior como na periferia. A RD, quando presente, foi classificada em não proliferativa ou proliferativa, e foi avaliada a presença de edema macular diabético.

A avaliação da retina descrita baseou-se no olho com alterações retinianas mais graves. Nos pacientes que apresentaram mais de uma alteração retiniana, foi descrita a alteração mais grave, ou a que mais ameaçava a visão.

De acordo com o protocolo de exame, em caso de impossibilidade de exame da retina por opacidade de meios, foi testada a presença de percepção luminosa do olho em questão. Caso o olho de exame impossível apresentasse ausência de percepção luminosa, não se prosseguiu com a investigação diagnóstica do olho em questão.

## RESULTADOS

Foram avaliados dados referentes a 138 pacientes atendidos. Estes pacientes tinham 51,3±18,0 anos de idade (média, desvio padrão), e 75 pacientes eram do sexo masculino (54,4%). Houve relato de diabetes por 49 pacientes (35,5%) e de hipertensão arterial por 67 pacientes (45,5%).

### Catarata

Apresentavam cristalino transparente em ambos os olhos (30,4%) 42 pacientes, e 50 apresentavam pelo menos um olho pseudofácico (36,2%). A catarata esteve presente em pelo menos um olho em 32 pacientes (23,2%). A catarata congênita foi diagnosticada em dois pacientes (ambos os olhos), e um paciente apresentava cristalino subluxado em ambos os olhos. Dois pacientes apresentaram afacia (um deles em ambos os olhos), e um paciente tinha uma prótese ocular em um dos olhos. Sete pacientes tinham o exame da câmara anterior impossível em pelo menos um dos olhos por opacidade de meios, *phthisis bulbi* ou glaucoma neovascular com desorganização da câmara anterior (um dos pacientes apresentou impossibilidade de exame em ambos os olhos).

### Avaliação da retina

Seis pacientes apresentavam impossibilidade do exame da retina por opacidade de meios e não tinham percepção luminosa nos olhos em questão. Esses pacientes foram excluídos das análises subsequentes. Outros três pacientes tinham o exame retiniano impossibilitado por opacidade de meios, mas apresentavam percepção luminosa e foram submetidos à ultrassonografia, que revelou retinas aplicadas e ausência de anormalidades detectáveis pelo método. Estes também foram excluídos das análises subsequentes.

Em relação aos 129 pacientes restantes, os achados fundoscópicos estavam distribuídos conforme a tabela 1.

**Tabela 1:** Alterações fundoscópicas em pacientes que aguardavam tratamento fora do domicílio em Rio Branco, Acre, abril de 2014

Alteração	Número de pacientes	Observação
Cicatriz coriorretiniana	6	-
Distrofia retiniana	3	-
Degeneração macular relacionada à Idade	5	1 caso com forma exsudativa e indicação de tratamento
Descolamento de retina regmatogênico	23	-
Pós-operatório de descolamento de retina	10	6 olhos com óleo de silicone
Glaucoma	14	1 caso de glaucoma congênito
Hemorragia vítrea	1	Não associada a diabetes
Maculopatia a esclarecer	4	-
Membrana epirretiniana	3	-
Neuropatia óptica isquêmica	1	Quadro sequelar
Nevus ou tumor de coroide	2	-
Exame normal	20	Destes, 8 pacientes com diabetes
Retinopatia diabética	32	Não Proliferativa:10 (destes, 5 com Edema Macular); Proliferativa: 22
Retinopatia hipertensiva	3	2 com oclusão vascular retiniana
Uveíte posterior	2	-

Total	129	

## DISCUSSÃO

Dos 138 pacientes que aguardavam TFD por doenças retinianas examinados, 40 tiveram a avaliação solicitada em função da RD (28,9%). Destes, 32 tinham alterações retinianas e 8 apresentaram exame da retina normal.

Em relação à avaliação do cristalino, 36,2% dos pacientes apresentaram pseudofacia em pelo menos um dos olhos. Este dado indica que uma parcela significativa dos pacientes tinha sido submetida a tratamentos oftalmológicos de alta complexidade, como é o caso da facectomia com implante de lente intraocular, apesar do número escasso de oftalmologistas na região.

Entre as doenças da retina, as duas principais causas de alterações retinianas foram descolamento de retina regmatogênico e a RD. Estes achados estão de acordo com dados da literatura, que também relataram as duas doenças como importantes causas retinianas de cegueira em adultos de populações urbanas.^(3)^

O descolamento de retina regmatogênico estava presente em 23 pacientes (17,8%). Dez pacientes encontravam-se no pós-operatório tardio de cirurgia para correção de descolamento de retina e necessitavam de acompanhamento com subespecialista em retina; 6 destes pacientes tinham óleo de silicone na cavidade vítrea e eram candidatos a nova cirurgia para a remoção deste.

A RD estava presente em 32 pacientes (24,8%), e 27 destes necessitavam de tratamento por RD proliferativa ou edema macular.

Outras doenças retinianas, como cicatrizes de coriorretinites, distrofias, degeneração macular relacionada à idade, maculopatias sem diagnóstico (“a esclarecer”), membrana epirretiniana, hemorragia vítrea, uveítes posteriores, oclusões vasculares retinianas, retinopatia hipertensiva e doenças da coroide estavam presentes em 29 pacientes.

A RD é uma doença evitável que pode ser acompanhada à distância nos estágios iniciais. O acompanhamento da RD desde fases mais precoces do *diabetes mellitus *ocasiona uma redução na necessidade de tratamento, que é dirigido às complicações.^(9)^ Dos pacientes que aguardavam TFD, 20 não apresentavam nenhuma alteração no exame da retina; 8 deles eram portadores de diabetes. A maculopatia diabética pode ser suspeitada em um paciente com diabetes que apresenta baixa de acuidade visual, e pacientes com essas características muitas vezes devem ser investigados com exames complementares, como angiofluoresceinografia e tomografia de coerência óptica, para a detecção de alterações subclínicas.^(10,11)^

Dos 40 pacientes avaliados em função da diabetes, 13 (32,5%) necessitavam apenas de acompanhamento (casos de RD ausente ou não proliferativa sem edema macular) e 27 (67,5%) pacientes necessitavam de tratamento em função de edema macular ou RD proliferativa.

O Brasil tem um número estimado de 11,9 milhões de pessoas com diabetes.^(12)^ Dados da literatura apontam que, após 15 anos de doença, aproximadamente 78% dos pacientes com diabetes apresentarão algum grau de RD.^(13)^

Da perspectiva de economia em saúde, estabeleceu-se que a prevenção da RD apresenta melhor relação custo-efetividade do que tratamento dessa complicação. Estudos mostram que o montante gasto para um paciente que apresenta fases avançadas da RD ultrapassa dez vezes o custo para o tratamento das fases iniciais; o custo aumenta junto da progressão da doença.^(14)^ Para exemplificar, pode-se citar um dado calculado para o sistema de saúde da Alemanha, em 2002, segundo o qual o montante que poderia ter sido economizado com a prevenção de fases avançadas da RD corresponde a 1,5% do total do gasto com Saúde.^(14)^

Na realidade brasileira, pelas grandes dimensões nacionais e pela má distribuição de especialistas, acredita-se que o custo seja ainda maior, levando-se em conta aqueles relacionados ao TFD.

O acompanhamento de pacientes com diabetes e retinopatia ausente ou nos estágios iniciais pode ser realizado por meio de estratégia de telemedicina, que apresentou um bom perfil custo-efetividade.^(13,15-18)^ Tal estratégia pode ajudar a suprir a demanda por subespecialistas em localidades remotas, fornecendo apoio na tomada de decisões terapêuticas e no acompanhamento da evolução do tratamento.

A telemedicina está elencada nas propostas para a oftalmologia brasileira estabelecidas pelo CBO.^(2)^ Na amostra de pacientes avaliada no presente estudo, todos os pacientes que aguardavam pelo TFD poderiam ter se beneficiado de uma opinião do especialista à distância. Desta forma, tanto pacientes com exame da retina normal poderiam readequar seu acompanhamento na própria região, evitando uma viagem, como os pacientes com doenças de tratamento mais complexo poderiam ter sua viagem e tratamento realizados com mais urgência.

Acreditamos que, com o intuito de reduzir os casos de cegueira evitável por doenças da retina em áreas remotas do Brasil, algumas medidas podem ser adotadas, como: (i) plano de carreira profissional para atrair e fixar o especialista em áreas remotas; (ii) investimento em equipamentos para diagnóstico e tratamento das doenças retinianas (retinógrafos, tomógrafos de retina, aparelho de fotocoagulação, centro cirúrgico etc.); (iii) capacitação e treinamento de oftalmologistas para diagnosticar e tratar doenças da retina; (iv) implantação de sistema de telemedicina para envio de imagens à distância para diagnóstico e consultoria por subespecialistas em centros de leitura; (v) capacitação e treinamento de profissionais para educação em saúde, e realização e envio de retinografias para a estratégia de telemedicina.

## CONCLUSÃO

As principais doenças retinianas nesta amostra de pacientes foram a retinopatia diabética e o descolamento de retina. A retinopatia diabética é uma causa de cegueira evitável que pode ser acompanhada à distância, nos estágios iniciais, por meio de estratégias de telemedicina. A telemedicina pode ser uma importante ferramenta no acompanhamento de doenças retinianas em localidades remotas no Brasil.
